# What Is *Global Health: Science and Practice* Doing to Address Power Imbalances in Publishing?

**DOI:** 10.9745/GHSP-D-20-00453

**Published:** 2020-09-30

**Authors:** Sonia Abraham, Stephen Hodgins, Abdulmumin Saad, Madeleine Short Fabic

**Affiliations:** a Scientific Editor, Global Health: Science and Practice Journal, Baltimore, MD, USA.; b Editor-in-Chief, Global Health: Science and Practice Journal, and Associate Professor, School of Public Health, University of Alberta, Edmonton, Alberta, Canada.; c Associate Editor, Global Health: Science and Practice Journal, Washington, DC, USA.

What is labeled “global health” has largely concerned the practice of public health work **elsewhere**, generally in low- and middle-income countries (LMICs).^1^ Indeed, global health’s key feature is that its power structures are generally located in high-income countries (HICs) while its implementation is generally located in LMICs. This imbalance is a result of colonial history, funding sources, and social and economic structures that have conferred power—including privilege, prominence, recognition, funding, opportunity, and decision-making authority—to institutions and individuals based in HICs. These deep-rooted structures have helped amplify the voices of those in HICs over the voices of those based in LMICs.^2^ In such a system, it is accepted that HICs have expertise to provide and LMICs have capacity gaps to fill.^3^ This imbalance is reflected in global health program planning, implementation, research, and publishing.^4^^,^^5^ We recognize that they are also reflected at GHSP.

Amplified voice for those based in United States and elsewhere in HICs and diminished voice for those based in LMICs is a poor recipe for improving well-being or strengthening institutions around the world.^6^ Indeed, the notion that HICs have something to “teach” LMICs but nothing to learn is a reflection of skewed perceptions of expertise and power. These asymmetries have grown even more evident during the COVID-19 pandemic.^7^

Recent efforts to “decolonize global health” signal an increasing commitment by many players to address these issues of imbalance and inequity.^8^ At GHSP, we recognize that to meaningfully engage in addressing power imbalances, as a first step, we must look at our own attitudes and practices. We are especially interested in identifying how we need to do things differently to reflect a range of voices and perspectives in our journal that better corresponds to where this work is actually being done.^9^

1. We completed a critical examination of the demographic composition of our editorial board and associate editorial team. We found that although we have some gender diversity among our editorial team and editorial board,^9^ institutions and individuals based in LMICs are underrepresented, as are people of color. We are actively working to ensure diversity in gender, race, ethnicity, and geography of our editorial board, associate editorial team, and peer reviewers.
2. We are committed to ensuring that perspectives from authors based in LMICs are evident in the articles we publish. Any article submitted to GHSP reporting results from research or program experience in specific LMICs should include authors from these countries. In instances where a submission lacks local authorship, under our revised authorship guidelines, the corresponding author is now ex-pected to provide reasons for any such omission in the cover letter accompanying the submission. Additionally, we are working to revise our instructions for authors to better promote ethical authorship practices.^10^
3. We are committed to removing publication barriers that can disproportionately impact authors based in LMICs. Recognizing that journal fees can be a major impediment to article submission and publication, especially for researchers in LMICs,^11^ we continue to make GHSP a no-fee, open-access journal. Furthermore, in instances where English language barriers could hinder publication opportunities, our editorial team has worked with authors to address language barriers and is committed to continuing this practice.
4. We expect that any individuals or institutions from HICs that are implementing programs or conducting research in LMICs will respectfully and substantively engage stakeholders from those countries in decision making, planning, implementation, and research and are revising our instructions to authors to reflect that expectation.^12^^,^^13^

We recognize that the challenges of power asymmetries and inadequate diversity are complex and multilayered, and our efforts to address them constitute only a modest first step in the needed direction. We commit to holding ourselves to account, and we invite our readers and other stakeholders to hold to us to account as we work to meaningfully follow through on these initial actions. To that end, we invite our readers, especially those in LMICs, to share their perspectives through letters to the editor, commentary, and research.
